# RDW-CV and Male Sex as Possible Response Factors to 9-Month Colorectal Cancer Palliative Chemotherapy

**DOI:** 10.3390/jcm14155201

**Published:** 2025-07-23

**Authors:** Maciej Jankowski, Ewelina Grywalska, Mansur Rahnama, Tomasz Urbanowicz

**Affiliations:** 1 Clinical Oncology and Immuno-Oncology Department with Day Outpatient Sub-Department and Reception Unit of Greater Poland Cancer Centre, Garbary Street, 61-866 Poznan, Poland; 2 Department of Experimental Immunology, Medical University of Lublin, 4a Chodźki Street, 20-093 Lublin, Poland; 3 Department of Dental Surgery, Medical University of Lublin, 6 Chodźki Street, 20-093 Lublin, Poland; 4 Cardiac Surgery and Transplant Medicine Department, Poznan University of Medical Sciences, Dluga ½ Street, 61-848 Poznan, Poland

**Keywords:** colorectal cancer, palliative therapy, male sex, CT scan

## Abstract

**Background/Objectives**: Colorectal cancer (CRC) is one of the major epidemiological oncological confronts with established risk factors, including male sex. Still, CRC is reported among the leading malignancies in the female population. The necessity for possible, easily accessible prognostic factors is required to improve patient outcomes. This study aimed to assess sex-related differences in nine-month four-stage CRC results of palliative systemic therapy. **Methods**: A total of 67 patients (39 males) with a median age of 70 (64–76) years were referred for first-line palliative chemotherapy due to end-stage colorectal cancer diagnosis. The CRC advancement was evaluated by computed tomography (CT) before and 9 months after chemotherapy. The demographical and clinical characteristics were evaluated for nine-month therapy outcomes, including mortality risk and CT scan results. **Results**: The nine-month mortality risk in female and male groups was indifferent, reaching 21% (6 patients) and 21% (8 patients), respectively (*p* = 0.935). Among survivors, therapy response was observed in 6 (21%) female and 20 (51%) male patients (*p* = 0.056). In multivariable analysis, the male sex (OR: 3.91, 95% CI: 1.09–14.05, *p* = 0.037) and RDW (OR: 0.61, 95% CI: 0.42–0.88, *p* = 0.008) were found to be significant for disease response to systemic therapy based on CT scan results. The ROC curve for predictive role yields a sensitivity of 71.1%, specificity of 57.8%, and an area under the curve (AUC) of 0.726. **Conclusions**: Our analysis points out the possible favorable role of the male sex on nine-month systemic therapy response in palliative CRC. The RDW-CV can be regarded as a possible indicator of chemotherapy response in colorectal cancer. The mortality risk within 9 months of systemic therapy is comparable between males and females.

## 1. Introduction

Colorectal cancer (CRC) is one of the major epidemiological oncological confronts worldwide [1]. Despite a growing body of research focused on early diagnosis and risk factors identification, the late onset is still one of the medical challenges related to inferior prognosis [2,3]. CRC represents one of the most common malignancies with established risk factors such as older age, male predominance, hereditary predisposition, physical inactivity, and excessive adipose tissue accumulation [4]. Up to one out of ten new CRC cases are linked with non-polyposis colorectal cancer, familial adenomatous polyposis, or other hereditary syndromes [5].

CRC is reported as one of the leading malignancies in the female population [6]. In recent years, the CRC mortality rate has been reported to decrease in males by 5% and female patients by almost 10% [7]. It is considered that estrogens may diminish colorectal cancer risk by modulating anti-tumor immune responses [8].

Metastatic CRC is a dissimilar disease requiring complex and dynamic management, including applied therapies, including triplet chemotherapy when the more aggressive approach is indicated [9]. Numerous possible prognostic factors were applied to improve patient outcomes, including simple inflammatory markers from peripheral blood count analysis [10,11,12].

The oncologic diseases are believed to induce chronic inflammatory activation [13]. Innate immunity may promote malignant progression [14]. The interleukin-6, tumor necrosis factor-alpha, and C-reactive Protein (CRP) were found to be related to tumor progression [15].

In recent studies [16], inflammation-related biomarkers obtained from peripheral blood count analysis have been proposed as useful predictors of worse outcomes. Next to identities such as neutrophil-to-lymphocyte ratio (NLR) and monocyte-to-lymphocyte ratio (MLR), an adverse relation between red cell distribution width (RDW) and CRC has been suggested [17].

The red cell distribution width (RDW) describes the heterogeneity of circulating red blood cells. Initially, this parameter was applied to diagnose anemia. Its utility in various pathological conditions’ mortality prediction has been noticed, including cardiovascular, pulmonary, gastrointestinal tract, and kidney diseases [18,19].

The clinical utility of the hemoglobin to RDW ratio in differential diagnosis of CRC from benign colorectal lesions has been postulated [20]. In the recent Korzinek et al. [21] study, the diagnostic value of higher levels of RDW was confirmed, differentiating CRC from adenomatous lesions.

In Peng et al.’s study [22], the RDW was related to increased postoperative complications risk, but not overall survival among CRC patients undergoing radical surgery. In contrast, Zhao et al. [23] in their analysis performed on 6.224 CRC patients postulated the prognostic value of preoperative higher RDW on overall and disease-free survival in CRC patients treated by radical resection in China. Wen et al. [24], in their meta-analysis, pointed out the differences regarding the RDW methodology on their prognostic value. As the RDW-coefficient of variation (RDW-CV) is a relative measure presented in percentages, the RDW standard deviation (RDW-SD) is an absolute measure given in femtolitres. The prognostic value of RDW-SD as an independent overall survival and disease-free survival (DFS) was identified; the RDW-CV was recognized as a DFS marker.

This study aimed to assess sex-related differences in the nine-month four-stage CRC results of palliative systemic therapy. We aim to point out possible predictors of systemic therapy in palliative CRC.

## 2. Materials and Methods

Our retrospective analysis included 67 (39 (%) males) with a median age of 70 (64–76) years referred for first-line palliative chemotherapy due to end-stage colorectal cancer diagnosis between 2022 and 2024 in the Clinical Oncology and Immuno-Oncology Department with Day Outpatient Sub-Department and Reception Unit of Greater Poland Cancer Centre. Only patients with a diagnosed 4th-grade CRC, defined by disease spread beyond the gastrointestinal system, including other organs as lungs, liver, or peritoneum, who were treated with the same protocol of systemic therapy within a nine-month period, were enrolled in the study.

The CRC advancement was evaluated by computed tomography (CT) before and 9 months after chemotherapy. The demographical and clinical characteristics were evaluated for nine-month therapy outcomes, including mortality risk and CT scan results.

The exclusion criteria included second- or third-line systemic therapy or requirement for systemic therapy changes within nine months due to intolerance/side effects or other reasons. Any missing data was regarded as exclusion criteria.

The analyzed group was divided according to sex differences, as presented in Table 1.

### 2.1. Bioethics Committee

This study was conducted in accordance with the Declaration of Helsinki and approved by the Institutional Review Board (or Ethics Committee) of Poznan University of Medical Sciences, Poznan, Poland (protocol code 405/24 from 19 June 2024, for studies involving humans.

### 2.2. Statistical Analysis

Since the data did not follow a normal distribution, continuous variables were reported as medians and interquartile ranges (Q1–Q3). Categorical data were presented as numbers and percentages. The Mann–Whitney test compared interval parameters between proximal and non-proximal groups. Categorical data were compared using a chi-square test of independence. Both univariate and multivariable models were used to predict the efficiency of chemotherapy protocols based on CT imaging. The multivariable model was assessed using the best subset method. The results were presented as odds ratio (OR) and 95% confidence intervals (95% CI). The receiver operator curve (ROC) was performed to check the accuracy of confirming multivariable analysis results in the prediction model. Statistical analysis was performed using JASP version 0.14.1 (University of Amsterdam, The Netherlands), with a significance level set at *p* < 0.05 (https://jasp-stats.org).

## 3. Results

**Figure 1:**
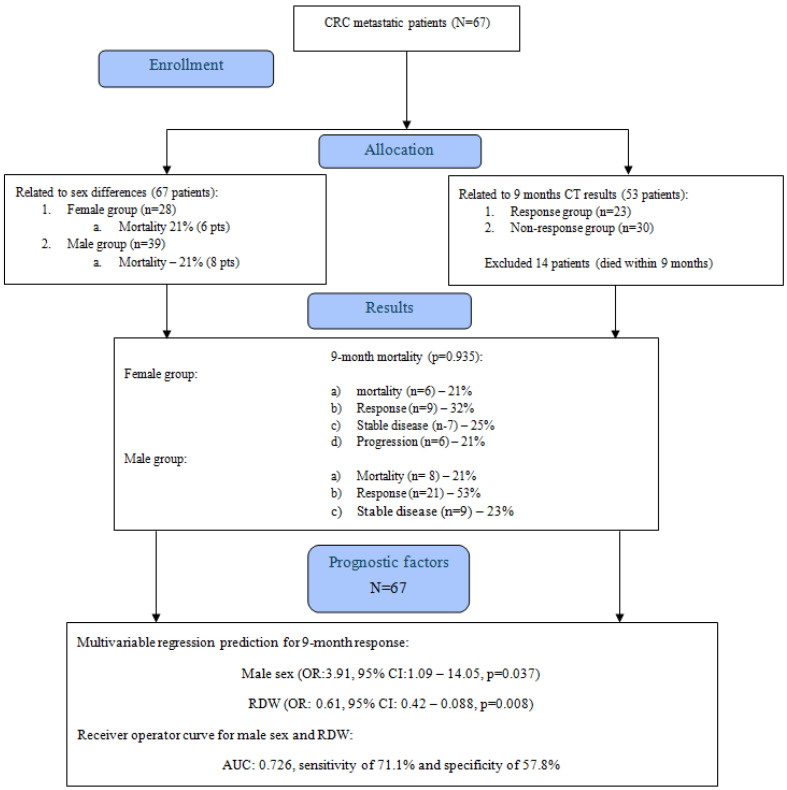
CONSORT flow diagram.

The nine-month mortality risk in female and male groups was indifferent, reaching 21% (6 patients) and 21% (8 patients), respectively (*p* = 0.935), as presented in Figure 1.

Fifty-three (79%) patients underwent surgical procedures before chemotherapy, including tumor resection (thirty-five (52%) patients) and hemicolectomy (eighteen (27%) patients). The female and male groups did not differ regarding the surgical intervention, as presented in Table 2.

### 3.1. Systemic Therapy

The systemic therapy was based on the patient’s clinical assessment and genetic results related to the presence of kras/nras/braf mutations. Standard therapy included the following: Folfox4 or Folfiri with or without EGFR inhibitors, Folfiri with Bevacizumab, Fluorouracil as monotherapy combined with folinic acid, capecitabine, or Irinotecan as monotherapy. EGFR-CTH therapy was based on CTH and anti-EGFR (cetuksymab or panitumumab) or VEGF-anti-angiogenic (bevacizumab) drugs according to National Comprehensive Cancer Network guidelines [25].

The standard dose was as follows:

FOLFOX4—oxaliplatin 85 mg/m^2^ on day 1.
leucovorin 100 mg/m^2^/day on days 1 and 2.
5-FU bolus 400 mg/m^2^/day followed by continuous infusion, repeated every fourth night with dose of 600 mg/m^2^/day on days 1 and 2, respectively.
Folfiri—Irinotecan 180 mg/m^2^ on day 1, leucovorin 100 mg/m 2/day on days 1 and 2, and 5-FU bolus 400 mg/m^2^/day followed by continuous infusion 600 mg/m^2^/day on days 1 and 2, repeated every 2 weeks.
Capecitabine 850–1250 mg/m2 orally twice daily for 14 days repeat every 3 weeks; monotherapy of irinotecan 180 mg/m^2^ was repeated every 2 weeks.
EGFR-CTH therapy was based on CTH (Folfox4 or Folfiri) and anti-EGFR (cetuksymab or panitumumab).
The anti-EGFR standard dose was: panitumumab 6 mg/kg given parenteraly on day 1 and repeated every 2 weeks.
Cetuximab 500 mg/m^2^ perenterally on day 1, and every fourth nights.
VEGF-CTH therapy was based on CTH (Folfox4 or Folfir) and anti-angiogenic drug bevacizumab—the standard dose of bevacizumab was 5–10 mg/kg on day 1, and repeated every 2 weeks.
Fluorouracil as monotherapy combined with folinic acid.

The maximal doses of the applied protocols as initial and after nine months of therapy were not statistically different within the time and between the analyzed groups, as presented in Table 2.

### 3.2. Survival Group

Among survivors, therapy response was observed in 6 (21%) female and 20 (51%) male patients (*p* = 0.056). A significantly higher partial response based on CT scan results was observed in the male group (*p* = 0.008). The 27% risk for disease progression in the female group compared to 3% in the male counterparts was noticed, as presented in Table 3.

### 3.3. Nine-Month Response Analysis

The group was subdivided according to CT scan imaging to identify possible clinical factors that may influence the 9-month palliative chemotherapy response. There were 53 patients with a median age of 69 (64–73) years who underwent further investigation, as presented in Table 4.

### 3.4. Uni- and Multivariable Analysis for Systemic Therapy Response

Uni- and multivariable regression analysis for possible predicting factors on nine chemotherapy responses in the analyzed group was performed. Demographical factors such as age, sex, and BMI were followed by co-morbidities (arterial hypertension and diabetes mellitus) and a family-positive history of oncology disease. Surgical intervention prior to the systemic therapy and EGFR-CTH therapy were analyzed, followed by laboratory findings as neutrophil to lymphocyte ratio (NLR), red cell distribution width (RDW), and serum creatinine.

In univariable analysis, the RWD-CV was found as a single predictive factor (OR: 0.64, 95% CI: 0.46–0.90, *p* = 0.011). In multivariable analysis, the male sex (OR: 3.91, 95% CI: 1.09–14.05, *p* = 0.037) and RDW (OR: 0.61, 95% CI: 0.42–0.88, *p* = 0.008) were found significant for disease response to systemic therapy (Table 5).

### 3.5. Receiver Operator Curve (ROC) for 9-Month Chemotherapy Response Predictors

**Figure 2:**
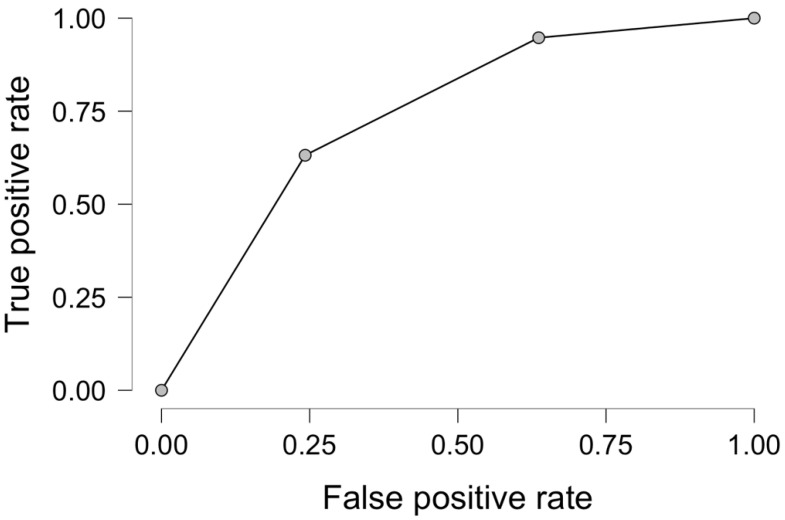
Receiver operator curve analysis for male sex and RDW-CV as predictive factors for 9-month response to systemic therapy in palliative CRC.

ROC analysis for nine months of response to chemotherapy in palliative CRC patients presented in multivariable regression analysis revealed male sex and RDW, yielding a sensitivity of 71.1%, specificity of 57.8%, and an area under the curve (AUC) of 0.726 as presented in Figure 2. The cut-off value for RD-CV in our predictive model was 14%.

## 4. Discussion

The results from our analysis point out possible predictive factors for systemic therapy response in palliative CRC. The male sex and RDW-CV were found to be significant. The study involved a relatively limited number of patients treated for nine months with chemotherapeutic protocols that were non-significantly modified throughout the analyzed period.

The recent publications [26] indicate a 5-year survival improvement in localized and regional CRC, suggesting the need for either therapy innovations or new prognostic factor definitions. A recent study by Hamers et al. suggested the impact of palliative CRC screening on their survival [26]. Xie et al. [27], in their analysis, revealed a novel BAP1-MAFF signaling axis that may be fundamental for CRC growth and could be regarded as either a prognostic marker or a potential therapeutic target. The possible prognostic role of liver metastatic involvement in disseminated CRC was proposed by Cohen et al. in the ARCAD pooled analysis [28].

The male sex is characterized by a higher incidence and inferior outcomes in CRC cancers [29]. Lifestyle, drinking habits, and gut microbiota can explain sexual dimorphism in CRC epidemiology [30].

Among other conditions, obesity is considered one of the known qualities allied to the male CRC population [31]. Sex differences may imply disease incidence, clinicopathological qualities, therapeutic tolerability, and outcomes [32]. Palliative chemotherapy was found in our analysis to be related to comparable mortality rates in both sexes. In previous reports, the higher risk for adverse effects of systemic therapy in females was postulated [33]. The obtained results were in coordination with previous reports [34]. The novelty of our analysis is based on male-sex-related favorable responses to applied therapy in four-stage CRC.

RDW is a simple and easily accessed parameter from peripheral whole blood count analysis that can be applied in clinical practice for CRC patients referred for systemic therapy. The clinical utility of RDW in breast tumor differentiative diagnosis was postulated by Lu et al. [35]. Nocini et al. [36], in their meta-analysis, presented the significance of RDW for survival prediction in laryngeal cancer patients. The relations between various pathological conditions and anisocytosis (reflected by RDW value) are widely postulated [37]. The RDW values may be modified by either malnutrition or acute infection [38]. In our analysis, none of the patients reported infection episodes either at the time of study enrollment or characterized by malnutrition. Cancerogenesis impairs erythropoiesis, resulting in RDW disturbances, by oxidative stress, peritumoral inflammatory activation, and poor nutritional status. Our analysis indicates an inverse relationship between chemotherapy response. The RDW-CV values can be explained by lesser degrees of hematological disturbances and abnormal red blood cell production. The heterogeneous red cell population results in impaired oxygen transportation, leading to peripheral tissue malfunction.

Chronic inflammatory system activation is considered one of the significant CRC drivers. Chronic inflammatory bowel diseases are considered potential risk factors of colitis-associated colorectal cancer (CA-CRC). It is believed that therapies targeting key inflammatory pathways and immune responses could lead to a reduction in CRC risk as part of personalized treatment in inflammatory bowel diseases [39]. In the report [40], the therapeutic response in CRC patients was suggested to be succeeded by cytotoxic lymphocytes and antigen-presenting macrophages modification by interferon. In Grellier et al. review [41], the involvement of gut microbia and the benefits of its potential modification on carcinogenic risk in inflammatory bowel disease was presented. Previous reports also postulated the link between inflammatory markers such as interleukin-8 and disease progression [42].

The accessibility to RDW is one of the strong points of our clinical practice analysis. The diagnostic utility of RDW in clinical practice regarding the tumor location was suggested in the Fancellu et al. study [43]. In the early stage of CRC, the propensity matching analysis performed by Cheng et al. [44] presented a negative association between RDW and patients’ survival. Next to higher levels of eosinophils and RDW, other parameters derived from whole blood count analysis, such as lower levels of hemoglobin, mean platelet volume (MPV), and mean corpuscular hemoglobin concentration (MCHC), were suggested as possible negative predictors in CRC patients [45].

The circulating microRNA as a possible predictor for systemic response in palliative CRC treatment was noticed in the Zhang et al. study [46]. Mhaidat et al. [47] pointed out the predictive value of protein 78 (GRP78) in colorectal cancer response to chemotherapy. The modulatory role of genetic variations on applied therapy in four-stage CRC was found in Zhang et al.’s analysis [48]. All those markers are sophisticated and highly accurate, but may be limited in everyday practice. Our analysis highlights the possible clinical utility of a simple and easily accessible marker, obtained from peripheral blood analysis, that can be applied in everyday oncological practice. The male patients with RDW-CV values below 14% can be regarded as a subgroup of CRC patients with a higher risk of unfavorable response to applied systemic therapy.

### Study Limitation

The retrospective features of the single-center analysis point out significant limitations requiring confirmation in large-volume prospective studies. The analysis was performed on the elderly population and included patients on various systemic therapies.

## 5. Conclusions

Our analysis points out the possible favorable role of the male sex on nine-month systemic therapy in palliative CRC. The RDW can be regarded as a possible indicator of chemotherapy response in colorectal cancer. The mortality risk within 9 months of systemic therapy is comparable between male and female sex. The further analysis is required on large-scale population to confirm the presented results.

## Figures and Tables

**Table 1:** A comparison of sex-related groups.

Parameters	Whole Group n = 67	Female Group n1 = 28	Male Group n2 = 39	*p* n1 vs. n2
Demographical:				
Age (years) (median (Q1–Q3)	70 (64–76)	72 (63–75)	69 (65–76)	0.770
BMI (kg/m^2^) (median (Q1–Q3)	25.0 (22.3–27.8)	23.2 (21.2–28.1)	26.6 (23.9–27.7)	0.036
Co-morbidities:				
Arterial hypertension (n (%))	42 (63)	18 (64)	24 (62)	0.826
Diabetes mellitus (n (%))	14 (21)	4 (14)	10 (26)	0.364
COPD (n (%))	2 (3)	0 (0)	2 (5)	0.506
Smoking (n (%))	5 (8)	2 (7)	3 (8)	0.944
Family oncological history (n (%))	13 (19)	5 (18)	8 (21)	0.887
Tumor staging:				
4th grade (n (%))	67 (100)	28 (100)	39 (100)	1.000
Right-sided	20 (30)	10 (38)	10 (26)	0.425
Left-sided	47 (70)	18 (64)	29 (74)	0.425
Metastases (n (%))				
Multiple	27 (40)	10 (36)	17 (44)	0.899
Lungs	26 (39)	12 (43)	14 (36)	0.290
Liver	55 (82)	19 (68)	36 (92)	0.269
Peritoneum	5 (8)	2 (7)	3 (8)	0.936
Nodes	13 (19)	4 (14)	9 (23)	0.560
Laboratory results—prior therapy				
WBC (mmol/L) (median (Q1–Q3)	7.1 (5.8–8.8)	6.3 (5.3–7.5)	7.7 (6.7–9.0)	0.005
Hb (mmol/L) (median (Q1–Q3)	12.9 (11.8–13.8)	12.3 (11.5–12.9)	13.7 (12.1–14.5)	0.001
Hct (%) (median (Q1–Q3)	39 (37–42)	37 (36–39)	41 (38–44)	<0.001
NLR (median (Q1–Q3)	3.1 (2.3–4.3)	3.0 (2.2–4.0)	3.2 (2.3–4.3)	0.636
RDW-CV (%) (median (Q1–Q3)	14.3 (13.3–15.7)	14.6 (13.7–15.7)	14.1 (13.3–15.5)	0.312
Plt (10 × 9/L) (median (Q1–Q3)	274 (216–316)	266 (212–316)	274 (218–316)	0.884
Creatinine (mg/dL) (median (Q1–Q3)	0.84 (0.71–0.96)	0.71 (0.62–0.83)	0.93 (0.81–1.07)	<0.001

Abbreviations: BMI—body mass index, COPD—chronic obstructive pulmonary disease, n1—group 1, n2—group 2, Hb—hemoglobin, Hct—hematocrit, NLR—neutrophil-to-lymphocyte ratio, Plt—platelets, RDW-CV—red cell distribution width—coefficient of variation, WBC—white blood count, Q—quartiles.

**Table 2:** Applied therapy in the analyzed group.

	Whole Group N = 67	Female Group n1 = 28	Male Group n1 = 39	*p* n1 vs. n2
Prior to surgical intervention (n (%)):	53 (79)	22 (79)	31 (80)	0.935
Resection (n (%))	35 (52)	14 (50)	21 (54)	0.763
Hemicolectomy (n (%))	18 (27)	8 (29)	10 (26)	0.981
Therapy:				
EGFR-CTH (n (%))	33 (49)	15 (54)	18 (46)	0.557
CTH (n (%))	30 (45)	12 (43)	18 (46)	0.796
VEGF (n (%))	4 (6)	1 (4)	3 (8)	0.635
Applied maximal doses of therapy:				
1. EGFR-CTH:				
Initial (% of maximal dose)	91 (87–96)	90 (85–96)	92 (90–97)	0.876
After 9 months	88 (76–99)	90 (82–99)	88 (77–99)	0.737
2. CTH:				
Initial	92 (88–98)	91 (87–97)	92 (86–98)	0.913
After 9 months	89 (80–98)	88 (78–99)	89 (81–96)	0.941
3. VEGF:				
Initial	91 (89–98)	92 (87–99)	90 (83–98)	0.746
After 9 months	88 (79–99)	90 (78–99)	87 (81–99)	0.589

Abbreviations: EGFR-CTH—epidermal growth factor receptor-cystathionine gamma-lyase n1—group 1, n2—group 2, n—number, VEGF—vascular endothelial growth factor.

**Table 3:** Nine months of chemotherapy results among female and male patients in CRC cancer.

	Whole Group N = 53	Female Group n1 = 22	Male Group n2 = 31	*p* n1 vs. n2
Therapy response (n (%))	30 (57)	9 (41)	21 (68)	0.056
Complete response (n (%))	4 (8)	3 (14)	1 (3)	0.167
Partial response (n (%))	26 (49)	6 (27)	20 (65)	0.008
Stable disease (n (%))	16 (30)	7 (32)	9 (29)	0.838
Disease progression (n (%))	7 (13)	6 (27)	1 (3)	0.010

**Table 4:** Response and non-response groups comparison.

Parameters	No-Response n = 23	Response n = 30	*p*
Demographical:			
Age (years) (median (Q1–Q3))	73 (64–78)	68 (62–73)	0.178
Sex (male (%))	10 (44)	21 (70)	0.056
BMI (kg/m2) (median (Q1–Q3))	24.7 (22.7–28.3)	25.1 (22.8–27.6)	0.650
Co-morbidities:			
Arterial hypertension (n (%))	16 (70)	17 (57)	0.347
Diabetes mellitus (n (%))	5 (22)	7 (23)	0.902
Tumor staging:			
4th grade (n (%))	23 (100)	30 (100)	1.000
Left-sided	16 (70)	24 (80)	0.522
Right-sided	7 (30)	6 (20)	0.522
Metastases (n (%))			
Multiple	12 (52)	15 (50)	1.000
Lungs	14 (61)	12 (40)	0.170
Liver	20 (87)	24 (80)	0.715
Peritoneum	3 (13)	2 (7)	0.642
Nodes	6 (26)	7 (23)	0.986
Family history (n (%)):	4 (17)	6 (20)	0.802
Pior surgery (n (%)):	17 (74)	26 (87)	0.249
Resection (n (%))	11 (48)	20 (67)	0.175
Hemicolectomy (n (%))	7 (40)	6 (20)	0.393
Laboratory results—prior therapy:			
WBC (mmol/L) (median (Q1–Q3)	6.86 (5.57–8.73)	7.43 (5.53–8.79)	0.676
Hb (mmol/L) (median (Q1–Q3)	12.6 (11.4–13.2)	13.1 (12.00–13.8))	0.108
Hct (%) (median (Q1–Q3)	38 (34–40)	40 (38–42)	0.080
NLR (median (Q1–Q3)	3.04 (2.22–4.00)	3.04 (2.22–4.30)	0.838
RDW-CV (%) (median (Q1–Q3)	15.5 (13.6–16.6)	13.7 (13.1–14.6)	0.008
Plt (10 × 9/L) (median (Q1–Q3)	256 (210–294)	271 (216–312)	0.628
Creatinine (mg/dL) (median (Q1–Q3)	0.75 (0.71–0.84)	0.92 (0.81–1.01)	0.007
Chemotherapy			
EGFR (n (%))	10 (44)	18 (60)	0.241
CTH (n (%))	13 (52)	10 (33)	0.097
VEGF (n (%))	0 (0)	2 (7)	0.499

Abbreviations: BMI—body mass index, COPD—chronic obstructive pulmonary disease, CTH—cystathionine gamma-lyase, EGFR—epidermal growth factor receptor, Hb—hemoglobin, Hct—hematocrit, NLR—neutrophil-to-lymphocyte ratio, Plt—platelets, RDW-CV—red cell distribution width coefficient variation, VEGF—vascular endothelial growth factor, WBC—white blood count, Q—quartiles.

**Table 5:** Uni- and multivariable analysis for response to chemotherapy in CRC patients.

	Univariable			Multivariable		
	OR	95% CI	*p*	OR	95% CI	*p*
**Demographical**				3.91	1.09–14.05	0.037
Age	0.97	0.92–1.03	0.310			
Male Sex	3.03	0.97–9.44	0.055			
BMI	0.94	0.82–1.09	0.418			
**Clinical**						
Arterial hypertension	0.57	0.18–1.80	0.339			
Diabetes mellitus	1.10	0.30–4.03	0.891			
Family history	1.21	0.29–5.01	0.788			
**Laboratory—prior therapy:**				0.61	0.42–0.88	0.008
NLR	1.02	0.74–1.42	0.886			
RDW	0.64	0.46–0.90	0.011			
Prior surgery	2.29	0.56–9.35	0.247			
EGFR-CTH therapy	1.95	0.65–5.87	0.235			

Abbreviations: BMI—Body Mass Index, CI—confidence interval, EGFR-CTH—epidermal growth factor receptor-cystathionine gamma-lyase, NLR—neutrophil-to-lymphocyte ratio, RDW—red cell distribution width.

## Data Availability

Data supporting the presented results will be available after a reasonable request for three years after publication by contacting the corresponding author.

## Abbreviations

The following abbreviations are used in this manuscript:

AUC	Area Under the Curve
BMI	Body Mass Index
CRC	Colorectal cancer
CRP	C-reactive Protein
CI	Confidence interval
COPD	Chronic Obstructive Pulmonary Disease
CT	Computed tomography
EGFR-CTH	Epidermal growth factor receptor-cystathionine gamma-lyase
Hb	Hemoglobin
Hct	Hematocrit
MLR	Monocyte-to-lymphocyte ratio
NLR	Neutrophil-to-lymphocyte ratio
Plt	Platelets
Q	Quartile
RDW	Red cell distribution width
ROC	Receiver Operator Curve
VEGF	Vascular Endothelial Growth Factor
WBC	White blood cell count
